# Identification of neural circuits controlling male sexual behavior and sexual motivation by manganese-enhanced magnetic resonance imaging

**DOI:** 10.3389/fnbeh.2023.1301406

**Published:** 2023-12-21

**Authors:** Lorena Gaytán-Tocavén, Alejandro Aguilar-Moreno, Juan Ortiz, Sarael Alcauter, Edwards Antonio-Cabrera, Raúl G. Paredes

**Affiliations:** ^1^Escuela Nacional de Estudios Superiores, Unidad Juriquilla, UNAM, Querétaro, Mexico; ^2^Instituto de Neurobiología, UNAM, Querétaro, Mexico

**Keywords:** MEMRI, neural circuits, sexual behavior, sexual motivation, manganese

## Abstract

**Introduction:**

Different techniques have been used to identify the brain regions that control sexual motivation and sexual behavior. However, the influence of sexual experience on the activation of these brain regions in the same subject is unknown. Using manganese-enhanced magnetic resonance imaging (MEMRI), we analyzed the activation of brain regions in the sexual incentive motivation (SIM) and the partner preference PP (tests) on weeks 1, 5, and 10 in male rats tested for 10 weeks. AIM. In experiment 1, we analyzed the possible toxic effects of 16 mg/kg of MnCl_2_ on male sexual behavior, running wheel, and motor execution. In experiment 2, subjects were tested for SIM and PP using MEMRI.

**Methods:**

In both experiments, a dose of 16 mg/kg (s.c) of chloride manganese (MnCl_2_) was administered 24 h before subjects were tested and placed immediately thereafter in a 7-Tesla Bruker scanner.

**Results:**

In experiment 1, the dose of 16 mg/kg of MnCl_2_ did not induce behavioral alterations that could interfere with interpreting the imaging data. In experiment 2, we found a clear preference for the female in both the SIM and PP tests. We found a higher signal intensity in the olfactory bulb (OB) in week 1 of the SIM test compared to the control group. We also found increased signal intensity in the socio-sexual behavior and mesolimbic reward circuits in the SIM test in week 1. In the PP test, we found a higher signal intensity in the ventral tegmental area (VTA) in week 10 compared to the control group. In the same test, we found increased signal intensity in the socio-sexual and mesolimbic reward circuits in week 5 compared to the control group. Cohen's *d* analysis of the whole brain revealed that as the subjects gained sexual experience we observed a higher brain activation in the OB in the SIM group. The PP group showed higher brain activation in the cortex and subcortical structures as they acquired sexual experience.

**Discussion:**

As the subjects gain sexual experience, more structures of the reward and socio-sexual circuits are recruited, resulting in different, and large brain activations.

## Introduction

Sexual behavior in rodents consists of a series of stereotype movements that, under appropriate hormonal conditions, lead to copulation. A receptive female produces chemosensory-relevant cues (pheromones) that attract a male, inducing mutual investigation and facilitating mating, which is crucial for the species' survival. Several brain regions are involved in the control of male sexual behavior: the olfactory bulbs (OBs), the amygdala (AMG), the bed nucleus of the stria terminalis (BNST), and the medial preoptic area (MPOA). These brain regions also process pheromones (Halpern and Martínez-Marcos, 2003; Restrepo et al., 2004). Along with the ventromedial hypothalamus (VMH), these areas form the socio-sexual behavior circuit (SBN) (O'Connell and Hofmann, 2011, 2012). There is another circuit important for motivated behaviors, including sexual behavior, which consists of areas associated with rewards, such as the ventral tegmental area (VTA) and the nucleus accumbens (NACC), which form the mesolimbic reward circuit (MRS) (O'Connell and Hofmann, 2011). These two brain circuits, the SBN and the MRS, integrate external and internal stimuli and are important for the adaptation and survival of the individual. The SBN and the MRS circuits form a large social decision-making circuit with homologies in different species. The details of how these circuits are activated during social behaviors are beyond the scope of the present study, and a detailed description can be found elsewhere (O'Connell and Hofmann, 2011, 2012; Ventura-Aquino et al., 2018).

Classical studies have used different techniques to identify the brain regions involved in the control of sexual behavior, including lesions, hormone implants, neuronal recording, early gene expression, silencing hairpin RNA (shRNA) in specific brain areas, and optogenetics, among others (reviewed in Ventura-Aquino and Paredes, 2020). The different techniques have advantages and limitations. For example, in some of them, the animals must be euthanized after displaying the behavior; in others, we only get information from a particular region. The development of magnetic resonance imaging techniques allows us to obtain information on brain regions and circuits at the same time, and more importantly, we can test the same subject at different times, evaluating possible brain changes related to experience in the same animal (Ventura-Aquino and Paredes, 2020).

Manganese-enhanced magnetic resonance imaging (MEMRI) is a technique that allows us to identify which neural structures and circuits are activated after a particular behavior (Silva et al., 2008). Manganese ions (Mn^2+^) move into neurons through voltage-gated calcium channels (Drapeau and Nachshen, 1984; Narita et al., 1990). It is transported anterogradely along axons and can cross synapses (Sloot and Gramsbergen, 1994; Tjälve et al., 1995; Pautler et al., 1998, 2003). Mn^2+^ transport across a synapse relies on presynaptic release and post-synaptic uptake; therefore, the amount of Mn^2+^ transported may change depending on the strength of connections (Tjälve et al., 1995; van der Zijden et al., 2008; Van der Linden et al., 2009). Therefore, it is possible to produce quantitative indices of Mn^2+^ movement through a neural system, obtaining information about the neural circuits underlying a particular behavior. For example, MEMRI has been used to evaluate sound-evoked activity in behaving animals (Yu et al., 2005; van der Zijden et al., 2008), brain structures activated by odors (Yu et al., 2005; van der Zijden et al., 2008; Lehallier et al., 2012), running wheel (Eschenko et al., 2010), and learning (Jackson et al., 2011; Gálosi et al., 2017). One of the main disadvantages of using manganese chloride (MnCl_2_) to evaluate changes using MEMRI is that, at high doses, the administration of MnCl_2_ is toxic, inducing unspecific effects that might alter the behavior studied. In a recent study, we evaluated the possible toxic effects of MnCl_2_ on female rats. We found that a 16 mg/kg dose does not affect sexual behavior, wheel ruining, or motor execution in a rotarod test (RT) (Aguilar-Moreno et al., 2022).

Sexual behavior has both appetitive (motivational) and consummatory components. The display of mounts, intromissions, and ejaculations easily identifies the consummatory aspects. Sexual motivation is defined as the mechanisms that activate, direct, and cause persistent behaviors directed toward a sexual incentive (Ågmo, 1999; Agmo, 2008). An incentive is a stimulus with rewarding properties, which produces an approach behavior (Agmo, 2008; Paredes, 2014). A variety of techniques are used to evaluate sexual motivation in rodents (Agmo, 2008; Paredes, 2014; Ventura-Aquino and Paredes, 2017; Ventura-Aquino et al., 2018), but one of the most consistently used is the sexual incentive motivation test (SIM). In this test, the subject can interact with two incentives with different characteristics presented simultaneously. The subject can smell, hear, and see the incentives, which are confined in cages, separated by wire mesh, and placed on the opposite side of the arena. Since the subject does not have physical contact with the incentives, we evaluate the time spent in the vicinity of the two incentives; for example, a sexually receptive female on one side of the box and a sexually experienced male on the opposite end (Agmo, 2008; Ventura-Aquino et al., 2018). In the partner preference (PP) test, the subject and the incentive physically interact and mate (Paredes and Vazquez, 1999; Agmo, 2008; Ventura-Aquino et al., 2018). In the SIM test, we can evaluate the intensity of approach behaviors to an incentive without the confounding variables associated with the execution of sexual behavior.

**Figure 1:**
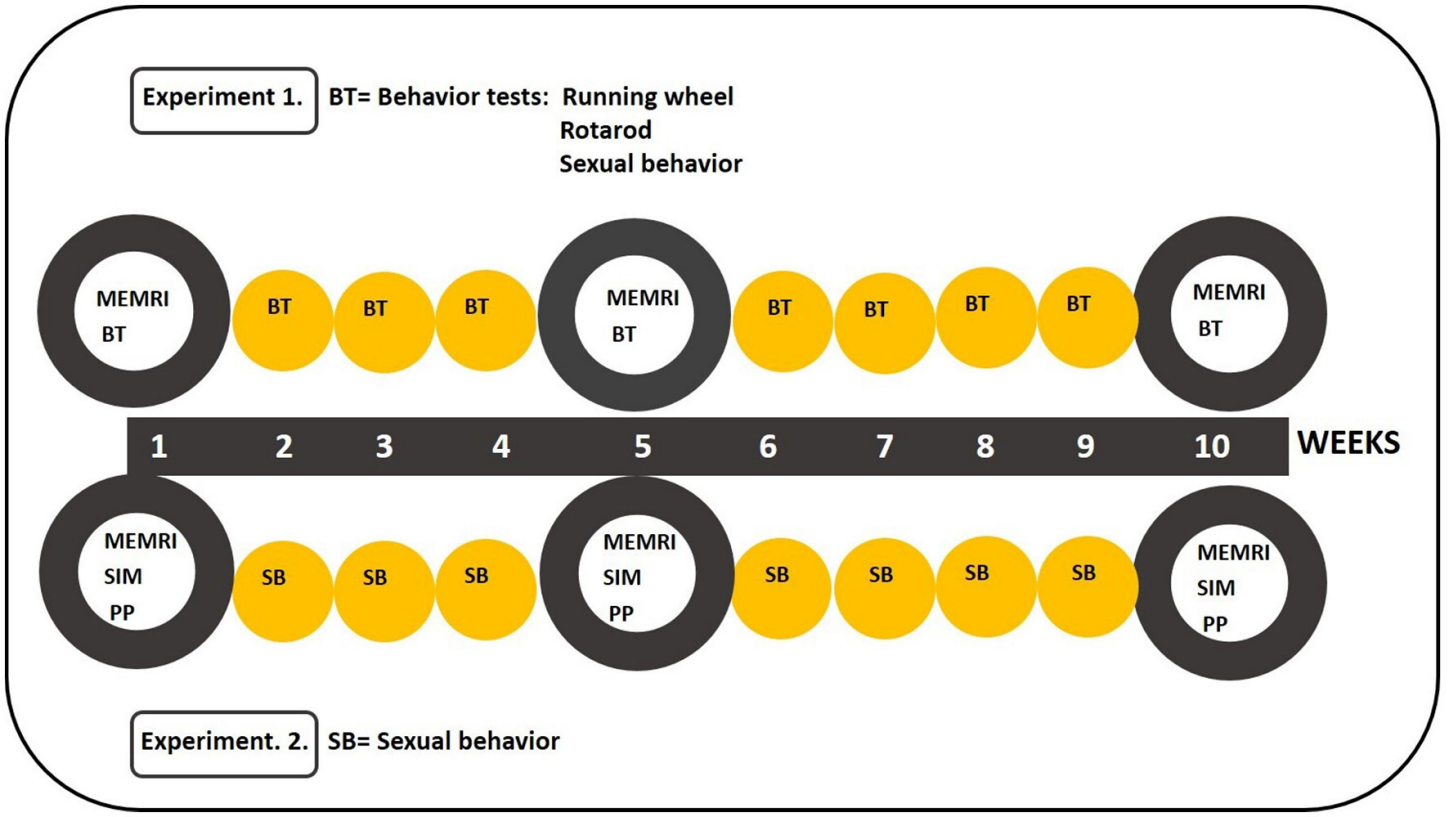
Schematic representation of the behavioral tests during the 10 weeks of experiments 1 and 2. In experiment 1, subjects were tested for sexual behavior, running wheel, and the rotarod every week. In experiment 2, subjects were tested for sexual incentive motivation (SIM) or partner preference (PP) on weeks 1, 5, and 10. They mated once a week the other weeks. Manganese chloride (MnCl_2_) was injected 24 h before the behavioral tests on weeks 1, 5, and 10 in both experiments.

In the present study, we performed two experiments. In experiment 1, we determined whether the 16 mg/kg dose of MnCl_2_ affected sexual behavior, running wheel, and motor execution in a rotarod test and if it produced an optimal signal to analyze magnetic resonance imaging in male rats. In the second experiment, we analyze the activation of brain areas in male rats after a partner preference (PP) or SIM test (see Figure 1) to determine if the consummatory and motivational features of sexual behavior activate different brain circuits (SBN and MRS) and if they are modified by sexual experience.

## Methods

### Experiment 1

The aim of experiment 1 was to evaluate the possible toxic effects of a dose of 16 mg/kg of MnCl_2_ on male sexual behavior, running wheel, and motor execution and determine if it induces an optimal MRI signal.

#### Subjects

Sexually naive male Wistar rats (300–350 g) from the animal care facility of the Instituto de Neurobiología were maintained in a reversed light/dark cycle (12/12). Since we wanted to evaluate how the activation of different brain regions and circuits is modified by sexual experience, our subjects had to be sexually naïve at the beginning of the experiment. They had free access to water and food in a temperature control room. They were randomly selected and assigned to a control group (*N* = 13) receiving saline or a group receiving a 16 mg/kg dose of MnCl_2_ (*N* = 14). The stimulus females of the same strain used in the behavioral test were ovariectomized and hormonally supplemented to induce sexual receptivity. They were treated with estradiol benzoate (25 mg/kg) and progesterone (1 mg/kg) for 48 and 4 h, respectively, before each behavioral test to induce sexual receptivity.

#### Sexual behavior tests

Sexually naïve males were placed in mating cages (40 cm × 60 cm × 40 cm) for 30 min with sexually receptive female rats. The following parameters were registered: the number of mounts (NMs), intromissions (NIs), and ejaculations (NEs); the mount (ML), intromission (IL), and ejaculation (EL) latencies; and the post-ejaculatory interval (PE).

#### Running wheel test

Before the experiment began, the males were trained in the running wheel (RW) in three sessions. The RW (LE904 (76-0412), Panlab-Harvard Apparatus^®^) was attached to a polycarbonate cage (42 × 26 × 19 cm, Allentown Caging Equipment). A multicounter (LE3806 Model) recorded the number of turns, and the SEDACOM 2.0 (Panlab-Harvard Apparatus^®^) was used to export the data to a computer. The distance traveled was calculated using the circumference of the wheel (diameter ^*^ π, 0.36 m ^*^ 3.14 = 1.13 m) multiplied by the number of wheel turns (1.13 m ^*^ # of laps) and presented in meters (m). Each session lasted 30 min, and males were tested once a week for 10 weeks immediately after the sexual behavior test.

#### Rotarod test

The rotarod test (IITC, Inc. Life Science, Series 8) assesses fine motor coordination. Males were habituated and trained in five sessions before testing. For testing, we programmed the apparatus in two different modalities. The rotarod rotated at 10 rpm for 180 s during the regular speed modality. In the increase speed modality, the rotarod started at 10 rpm, with the speed increasing until 15 rpm at 120 s, and the final 60 s maintained at 15 rpm. The fall latency was registered, and the test ended. The subjects were tested once weekly.

#### Behavioral testing procedure

The subjects were tested once a week for 10 weeks. They were tested for sexual behavior for 30 min, followed by RW for 30 min, and then the rotarod test. Twenty-four hours before weeks 1, 5, and 10, males were injected with saline or 16 mg/kg of MnCl_2._ All behavioral tests were performed under dim red light during the dark phase of the cycle (7 a.m. to 7 p.m.) starting at 10 a.m.

#### Manganese chloride administration

Manganese chloride (MnCl_2_; manganese (II) chloride SIGMA-ALDRICH, Product number 244589, St. Louis) was dissolved in saline (MnCl_2_ mg/kg/10 ml) and was injected subcutaneously (s.c) in a dose of 16 mg/kg. In all experiments, MnCl_2_ was injected 24 h before the behavioral evaluation in weeks 1, 5, and 10. We selected the dose from a previous study in which the same quantity had no toxic effects on the same behaviors in female rats (Aguilar-Moreno et al., 2022). We tried three doses in that study, including 8, 16, and 32 mg/kg of MnCl_2._ We stopped using the dose of 32 mg/kg because some of the subjects showed a reduction in different behavioral tests and developed skin lesions on the injection site. In the same study, we found that the best dose without toxic effects and the best image resolution is 16 mg/kg in a volume of 10 ml/kg.

### Experiment 2

Once we determined that a dose of 16 mg/kg of MnCl_2_ does not affect sexual behavior, running wheel, or motor execution, in experiment 2, we tested what structures were activated after sexual incentive motivation and partner preference tests and if this activation varies with sexual experience.

#### Subjects

Animals were maintained in the same conditions as described in experiment 1. Those males who ejaculated in three tests of sexual behavior with receptive females were selected for the experiment. They were randomly assigned to one of the following groups: the control group (*N* = 8) was left in their home cage; a group tested for sexual incentive motivation (*N* = 10) or a group tested for partner preference (*N* = 10).

#### Sexual incentive motivation

Sexual incentive motivation (SIM) was evaluated in a black acrylic arena (100 × 50 cm). The incentive animals, a sexually experienced male and a sexually receptive female, were placed in cages at diagonally opposed corners on each long wall. An opening (25 × 25 cm), located at floor level, communicated with each incentive animal cage as previously described (Ågmo, 2003; Portillo and Paredes, 2004). The experimental subject could hear, see, and smell the incentive animals, but no sexual contact was possible because a wire mesh separated them. A zone (20 × 30 cm) outside each incentive animal cage was designated the incentive zone. Before the experiment, the subjects were habituated to the testing environment during three sessions of 10 min each. During the habituation procedure, the incentive animals were not present. Before each session, the arena and the incentive animal cages were cleaned with a 0.1% solution of glacial acetic acid in water. The test lasted 10 min, and we recorded the time the experimental rats spent in each incentive zone. We evaluated SIM in weeks 1, 5, and 10. In the other weeks, males mated with receptive females in the mating cages. The subjects from the control group were always placed in the mating cage without stimulus animals.

#### Partner preference test

We evaluated partner preference in a three-compartment box made of wood. The middle compartment communicated with the lateral compartments through a sliding door (10 × 10 cm). A sexually receptive female and a sexually experienced male were placed in opposite lateral compartments. The stimulus animals wore a harness attached to the rear of the compartment with flexible rope, allowing them to display sexual behavior without leaving their compartment. During the test, the males were placed in the middle compartment. After 1 min, we removed the sliding doors, and the subjects were allowed to interact for 15 min. We quantified the time spent in each compartment and the different copulatory parameters. PP was tested once a week for 10 weeks, and the control group was always placed in the PP arena without the incentive animals. In weeks 1, 5, and 10, the subjects were placed in the scanner immediately after the test.

#### MnCl_2_ administration and behavioral testing procedures

Twenty-four hours before the SIM or PP tests, the subjects were injected as described in experiment 1 on weeks 1, 5, and 10.

#### MR image acquisition

Immediately after the behavioral test, we took the males to an adjacent room for scanning. The acquisition of images was performed in weeks 1, 5, and 10, which allows us to evaluate the changes in brain activation associated with sexual experience. Twenty-four hours before the behavioral tests, the males received a subcutaneous injection of MnCl_2_ at a dose of 16 mg/kg. We used a 7-Tesla Bruker scan (BioScan Bruker^®^ PharmaScan) to acquire high-resolution T1-weighted images with a fast low-angle shot (FLASH) sequence, TR = 50 ms, TE = 8 ms, flip angle = (20°), in-plane resolution = 200 mm, slice thickness = 200 mm, covering the whole brain and cerebellum. The animals were anesthetized with a 4% isoflurane–air mixture and placed in the scanner holder, where anesthesia continued with isoflurane at 2%. The respiratory and cardiac frequencies were monitored for the whole MR session.

#### MEMRI image preprocessing

Images were processed using FMRI Software Library v6.0 (FSL) and Advanced Normalization Tools (ANTs, v2.1) software libraries. We used the function *DenoiseImage* for denoising. Using the multivariate template construction tool (ANTs) (Avants et al., 2011), we created a template of the T1-weighted image brain average from the set of scans from week 1. After making the template, spatial normalization was performed on denoised images using the ANTs Registration SyN tool, and then the scans from weeks 1, 5, and 10 were registered to the template. We then used the tool *fslmerge* from the FMRIB Software Library v6.0 to merge all the aligned scans. Images with artifacts or bad corregistration were eliminated.

#### Image analysis

For the image analysis, we used the Paxinos and Watson (2007) rat atlas as a guide for drawing the template of different regions of interest (ROIs). We analyzed the ROIs from the olfactory bulb (OB), the bed nucleus of the stria terminalis (BNST), the medial preoptic area (MPOA), the ventromedial hypothalamus (VMH), the nucleus accumbens (NACC), the amygdala (AMG), the striatum (STR), the ventral tegmental area (VTA), and the hippocampus (Hipp). A ROI was created from the Harderian gland using the fslmaths tool of FSL for signal normalization. We tested for significant differences using the general linear model (GLM) implemented with the randomized tool in FSL using *t*-tests (Winkler et al., 2014) for comparisons between control and experimental groups (PP and SIM), with image thresholds at *Z*, min 0.95, and max 1. This analysis allowed us to evaluate the voxels' differences in intensity between groups in weeks 1, 5, and 10 in the different ROIs.

### Statistical analysis

Experiment 1. Data from the running wheel, rotarod, and sexual behavior tests were not normally distributed. Therefore, they were analyzed within groups by a Friedman repeated-measures ANOVA and Tukey as a *post-hoc* test. The analyses between groups were performed with a Mann–Whitney *U*-test. The percentage of mounts, intromissions, and ejaculations was analyzed with Fisher's exact test.

Experiment 2. The SIM and PP test data showed homogeneity of variance and normality and, therefore, were analyzed with parametric tests. The time spent with the stimulus male or female in the SIM and PP tests in weeks 1, 5, and 10 was analyzed by a two-way repeated-measures ANOVA and *post-hoc* Tukey's test. Sexual behavior parameters were analyzed as in the experiment 1.

Voxel intensity was determined by the FSL program (FMRI Software Library v6.0), and the data were analyzed by a two-way repeated-measures ANOVA and *post-hoc* Tukey's test. In a second analysis, we compared between groups' average signal intensity of the areas associated with the socio-sexual behavior circuit, including the OB, the VMH, the MPOA, the BNST, and the AMG. We did a similar analysis with the structures of the reward circuit, including the NACC, the Hipp, the striatum (STR), and the ventral tegmental area (VTA).

In a third analysis, we determined the Cohen's size effects (threshold 0.2–1.6) of the whole brain in weeks 1, 5, and 10, comparing the control and experimental groups and between weeks within the same group (SIM and PP). This analysis allows us to determine the effect size between the control and the experimental groups in weeks 1, 5, and 10. We obtained the group means from weeks 1, 5, and 10. For analysis between weeks, the mean of week 1 was subtracted from the mean of week 10 and then divided by the standard deviation of both weeks. The same procedure was applied for week 5, subtracting the mean of week 1 and dividing the result by the standard deviation of both weeks. This was also carried out to compare weeks 5 and 10. For the comparisons between the control and the experimental groups each week, we obtained the group mean from the control and each experimental group. Then, the mean of the control group was subtracted from the mean of the experimental groups (SIM or PP) and then divided by the standard deviation of both groups. Then, a voxel-wise analysis was performed on denoised, spatial, and signal-intensity normalized images. Using the *Glm* tool from FSL, contrasts for group comparisons were created, and the *randomize* function was used in the analysis. *p*-value-corrected images were binarized using *fslmaths* using the threshold of 0.95. Finally, the binarized images were corrected with Cohen's *d* analysis results.

## Results

### Experiment 1

#### Running wheel

**Figure 2:**
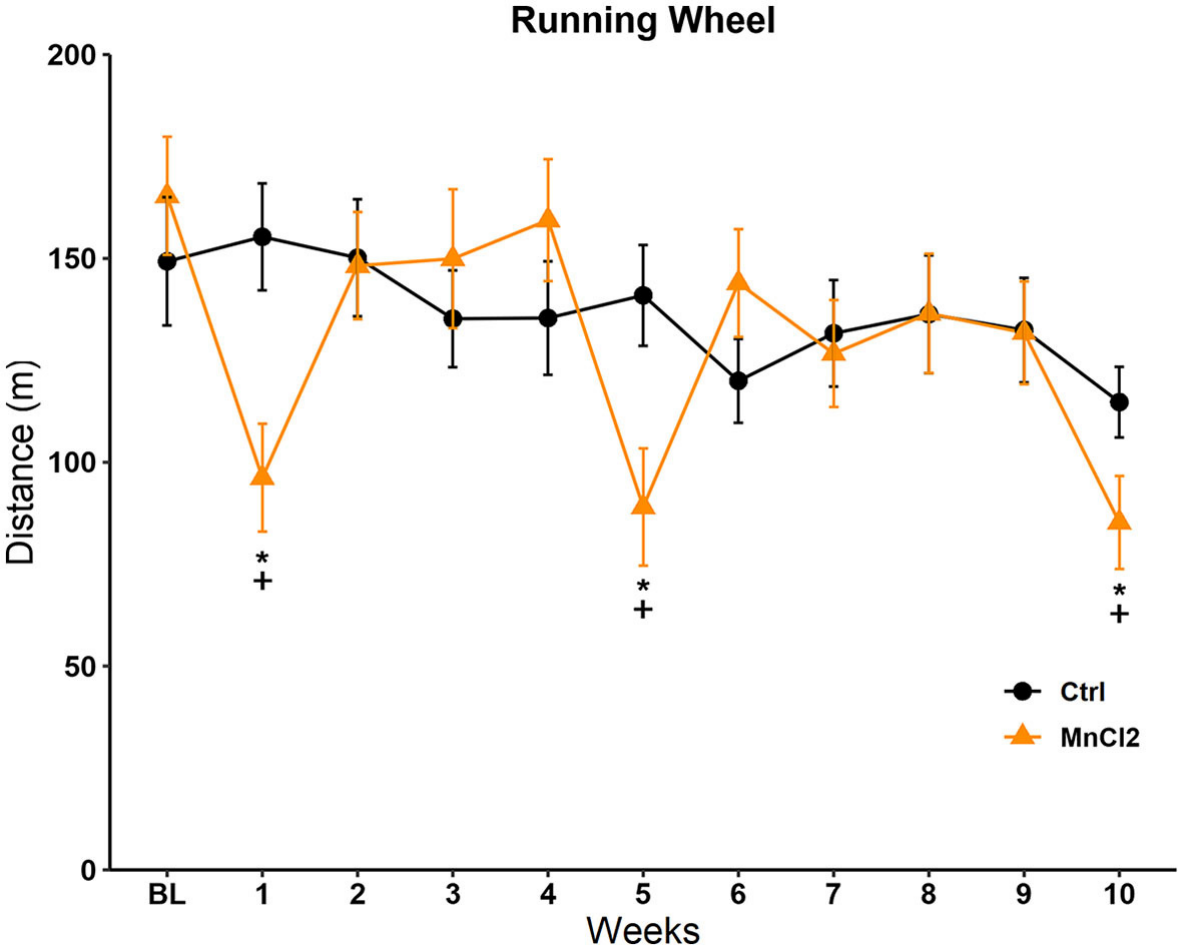
Distance traveled in the running wheel in subjects tested once weekly for 10 weeks. Male rats were treated with saline (*N* = 13) or manganese chloride (MnCl_2_; *N*= 14). Data are expressed in meters as mean ± SEM. *Different from baseline (BL) in the same group, *p* < 0.05. ^**+**^Different from the control group in the same session, *p* < 0.05.

The distance traveled on the running wheel during the 10 weeks of testing is depicted in Figure 2. The Friedman repeated-measures ANOVA revealed significant differences in the control [*X*^2^(10, *N* = 13) = 23.130; *p* = 0.01] and experimental groups [*X*^2^(10, *N* = 14) = 60.348; *p* < 0.001]. The *post-hoc* Tukey's test revealed that in the control group, there was a significant reduction in the distance traveled on week 10 (*q* = 4.683; *p* < 0.05) in comparison to week 1. In the experimental group, Tukey's test revealed significant differences against the baseline on sessions 1 (*q* = 5.077; *p* < 0.05), 5 (*q* = 6.326; *p* < 0.05), and 10 (*q* = 6.970; *p* < 0.05). The comparison between groups with the Mann–Whitney *U-*tests revealed a reduction in the distance traveled in the experimental group in contrast to the control group in sessions 1 (*U* = 31.500; *p* = 0.004), 5 (*U* = 42.000; *p* = 0.019), and 10 (*U* = 49.500; *p* = 0.046); that is, when MnCl_2_ was administered, see Figure 2.

#### Rotarod test 10 rpm

**Figure 3:**
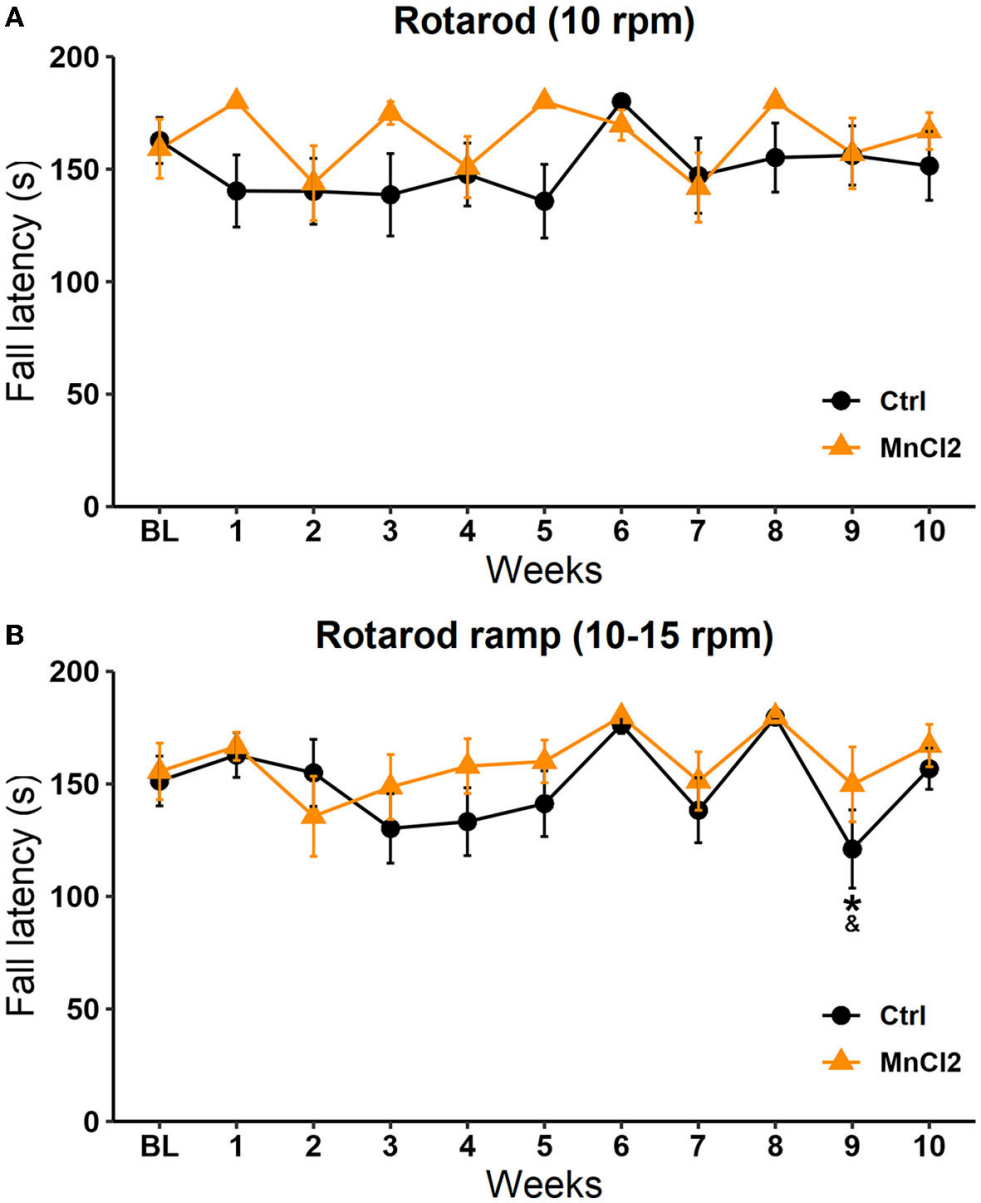
Fall latency in the motor coordination test in subjects tested weekly for 10 weeks. Male rats were treated with saline (*N* = 13) or with manganese chloride (MnCl_2_; *N* = 14) in two modalities: **(A)** at constant speed and **(B)** in ramp mode. Data are expressed in seconds as mean ± SEM.

The Friedman repeated-measures ANOVA revealed no significant differences between the control group [*X*^2^(10, *N* = 13) = 8.044 *p* = 0.625] and the experimental group [*X*^2^(10, *N* = 14) = 18.245 *p* = 0.051]. When we compared the latency to fall between groups, the Mann–Whitney *U*-test revealed a significant increase in the experimental group with respect to the control group on sessions 1 (*U* = 56.000; *p* = 0.014) and 5 (*U* = 56.000; *p* = 0.013), see Figure 3A.

#### Rotarod test 10–15 rpm

Significant differences along the sessions were observed in the ramp modality (10–15 rpm) in the control group [*X*^2^(10, *N* = 13) = 32.337; *p* < 0.001] but not in the experimental groups [*X*^2^(10, *N* = 14) =13.678; *p* = 0.188] according to the Friedman repeated-measures ANOVA. In the control group, the fall latency in week 9 was reduced in comparison to week 6 (*q* = 4.683; *p* < 0.05) and week 8 (*q* = 4.850; *p* < 0.05); see Figure 3B. These results suggest that the subcutaneous injection of MnCl_2_ does not affect fine motor coordination, which could interfere with the display of sexual behavior, SIM, PP, and the interpretation of the image analysis.

#### Sexual behavior

When the sexual behavior data were analyzed with Friedman repeated-measures ANOVA, a significant effect on the number of mounts was observed in the control group [*X*^2^(9, *N* = 13) = 15.056 *p* < 0.001]. A *post-hoc* test revealed a significant decrease in week 5 vs. 1 (*q* = 4.585; *p* < 0.003) and in week 10 vs. 1 (*q* = 4.427; *p* < 0.005). We also observed a significant difference in mount latency [*X*^2^(9, *N* = 10) = 26.141 *p* < 0.002]. *Post-hoc* tests revealed that the mount latency was significantly shorter in session 5 compared to session 1 (*q* = 6.189; *p* > 0.001) and in session 10 vs. session 1 (*q* = 5.605; *p* > 0.05). We also found a significant effect on intromission latency in the control group [*X*^2^(8, *N* = 10) = 18.985 *p* < 0.015]. The *post-hoc* Tukey's test revealed a decrease in the intromission latency in session 5 in comparison to session 1 (*q* = 5,485; *p* > 0.003) and in session 10 with respect to session 1 (*q* = 4.388; *p* > 0.049). Data are summarized in Table 1. No significant differences were found in other parameters; see Supplementary Table 1.

**Table 1:** Sexual behavior parameters from subjects in experiment 1.

**Groups**	**Control (*****n*** = **13)**			**MnCl**_**2**_ **(*****n*** = **14)**		
**Sessions**	**1**	**5**	**10**	**1**	**5**	**10**
**Percentage**						
Mount	79	100	100	73	100	100
Intromission	69	100	100	73	100	100
Ejaculation	61	100^*^	100^*^	36	73	91
**Number**						
Mounts	10.3 ± 1.7	3.9 ± 1.0^*^	3.3 ± 0.7^*^	12.9 ± 2.9	12.7 ± 3.2	5.9 ± 1.3^*#^
Intromissions	13.3 ± 0.9	9.3 ± 1^*^	9.6 ± 1.2^*^	10.1 ± 2.0	12.9 ± 1.6	10.8 ± 0.6
Ejaculations	2.4 ± 0.4	2.9 ± 0.2	2.8 ± 0.2	1.5 ± 0.3	2.3 ± 0.3	2.4 ± 0.2
**Latencies**						
Mounts	156.1 ± 70	6.8 ± 4.6^*^	43.9 ± 10.6^*^	154.5 ± 45.7	185 ± 58.3	34.2 ± 2.5^*#^
Intromissions	223.7 ± 30	14.8 ± 9.4^*^	80.7 ± 24^*^	328.4 ± 135	391 ± 128.3^+^	105.9 ± 42^*#^
Ejaculations	563 ± 108	314 ± 54.3	340 ± 54	1,131 ± 155^+^	528.3 ± 86.5	480.4 ± 43.1
Post-ejaculatory interval	362.5 ± 16	361.3 ± 4.1	374 ± 22.8	445.5 ± 79.5	444.4 ± 34	443.3 ± 35.3

Male rats were treated with saline (N = 10) or manganese chloride (MnCl_2_; N = 10) in weeks 1, 5, and 10. Data are expressed as mean ± SEM.

^*^Different from week 1 in the same group. p < 0.05.

^#^Different from week 5 in the same group. p < 0.05.

^+^Different from the control group in the same week. p < 0.05.

In the experimental group, we found a significant difference in the number of mounts [*X*^2^(9, *N* = 14) = 11.098 *p* < 0.05]. The *post-hoc* test revealed a statistically significant decrease in week 10 vs. week 5 (*q* = 4.557; *p* > 0.049) and week 1 (*q* = 4.557; *p* > 0.04). We also found significant differences in mount latency [*X*^2^(9, *N* = 10) = 24.586 *p* < 0.003]. The *post-hoc* test revealed a significant decrease in week 10 vs. week 5 (*q* = 4.857; *p* > 0.02) and week 1 (*q* = 6,557; *p* > 0.049). A similar reduction was observed in intromission latency [*X*^2^(9, *N* = 10) = 66.756 *p* < 0.003]. There was a significant decrease in week 10 vs. week 5 (*q* = 14.494; *p* > 0.049) and week 1 [*X*^2^(9, *N* = 10) = 34.536 *p* < 0.003]. No significant differences were found in other parameters; see Supplementary Table 1.

Throughout a Mann–Whitney rank sum test, we analyze the data between groups. We found an increase in intromission latency on week 5 in the MnCl_2_ group vs. the control group (MWU = 18, *T* = 117, *p* = 0.026). We found a significant increase in ejaculation latency on week 1 in the MnCl_2_ group compared to the control group (MWU = 7, *T* = 68, *p* = 0.022). No significant differences were found in other parameters or groups; see Supplementary Table 2.

### Experiment 2

#### Sexual incentive motivation test

**Figure 4:**
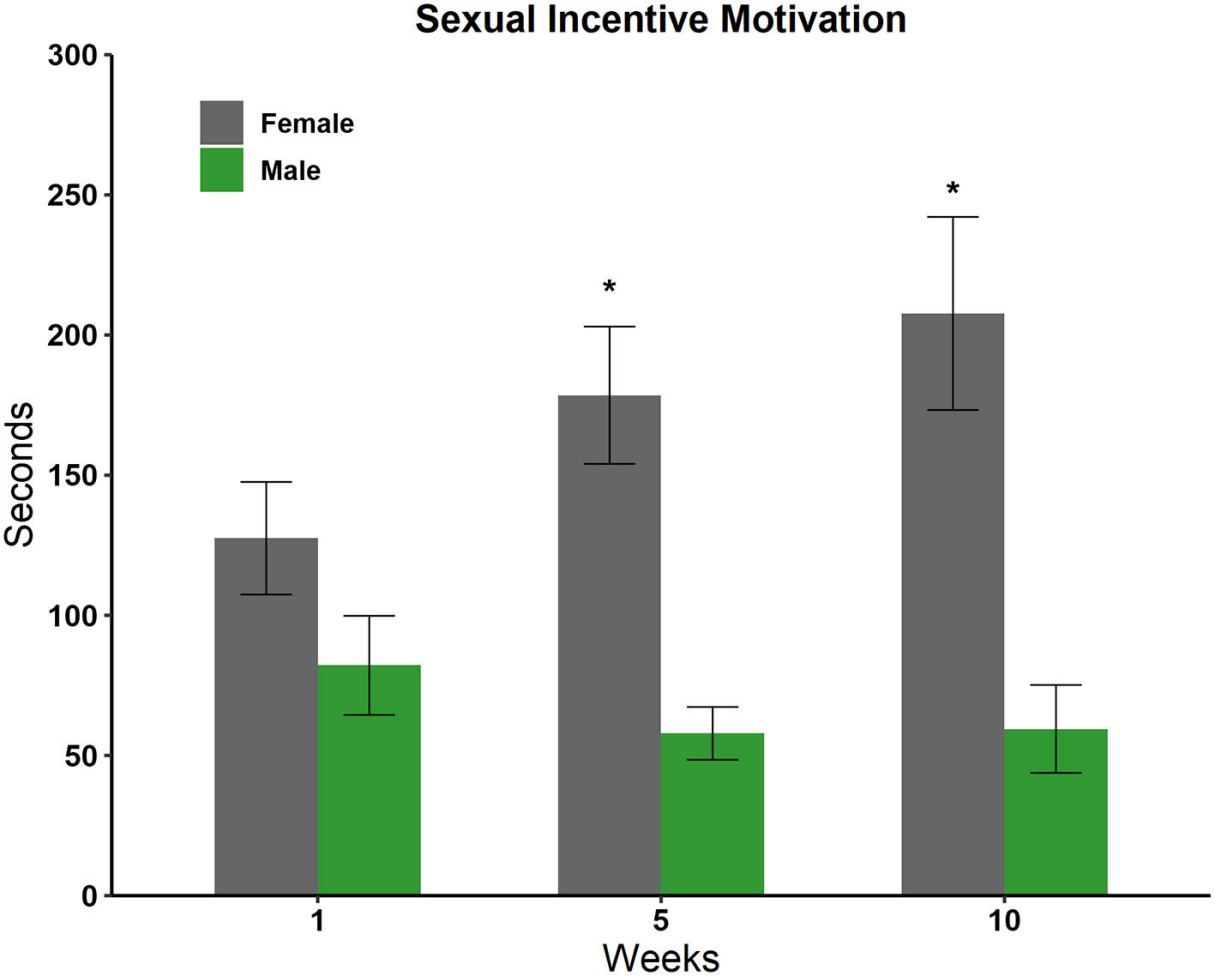
Time spent in the incentive zone of the male and female stimulus animals in weeks 1, 5, and 10 in the sexual incentive motivation test (*N* = 10). Data are expressed in seconds as mean ± SEM. *Significantly different from the time spent with the male; *p* < 0.05.

We compared the time spent in each incentive zone in weeks 1, 5, and 10 using a two-way repeated-measures ANOVA and Tukey as a *post-hoc* test. We observed a significant effect for sex [*F*_(1, 59)_ = 27,360 *p* < 0.001] and in the interaction sex by weeks [*F*_(2, 59)_ = 2,768 *p* = 0.05]. The *post-hoc* test revealed an increase in the amount of time that males spent in the female incentive zone in comparison with the time spent in the male incentive zone in weeks 5 (*q* = 25,182, *p* < 0.001) and 10 (*q* = 26,367, *p* < 0.001), see Figure 4.

#### Partner preference test

**Figure 5:**
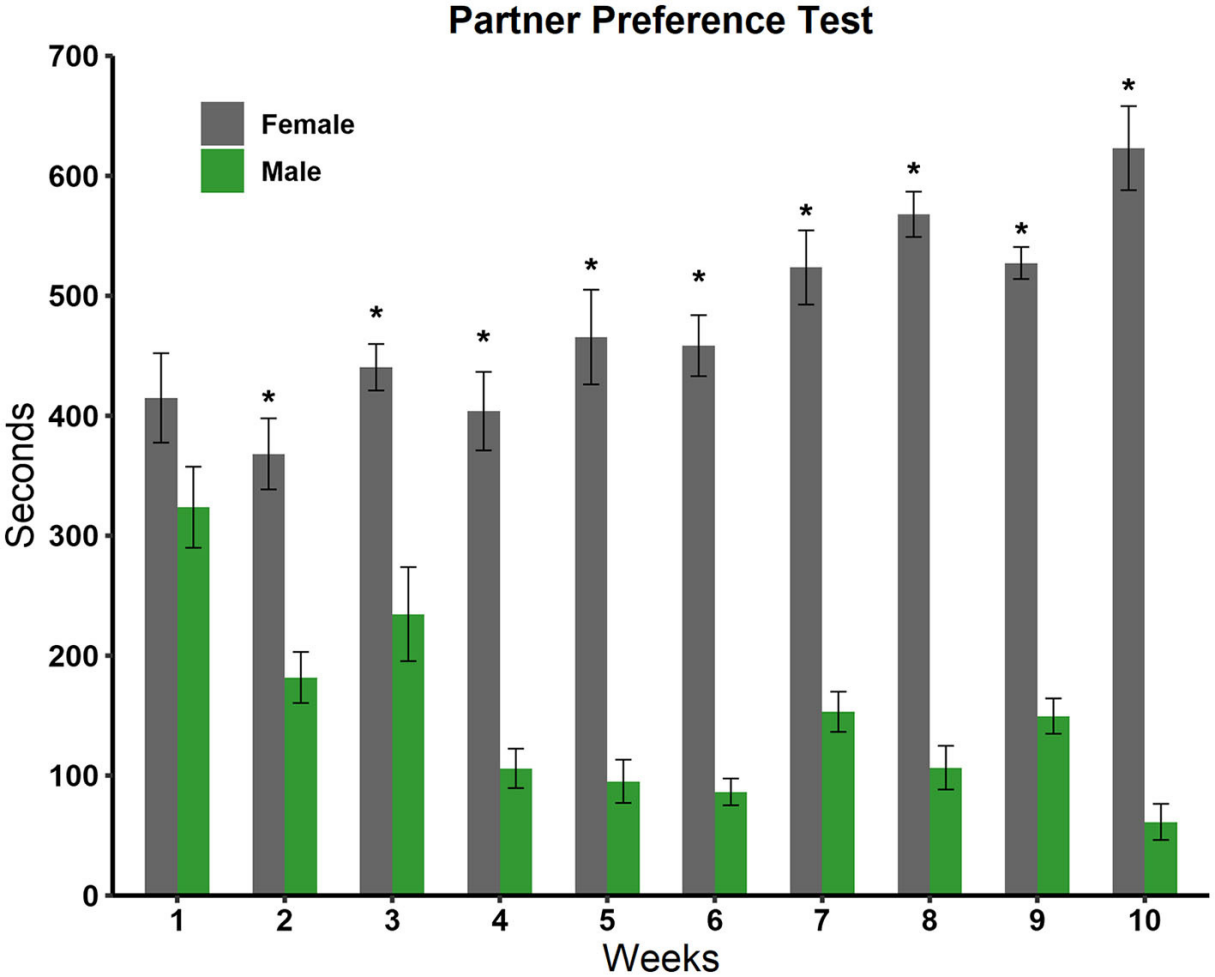
Time spent in the male and female stimulus animal compartments during the 10 weeks of testing in the partner preference test (*N* = 10). Data are expressed in seconds as mean ± SEM. *Significantly different from the time spent in the male compartment; *p* < 0.05.

We analyzed the time the experimental rats spent in the male and female compartments with a two-way ANOVA. We observed a significant effect in the sex factor [*F*_(1, 179)_ = 475,345 *p* < 0.001], in the weeks factor [*F*_(8, 179)_ = 5,495 *p* < 0.001], and in the interaction sex by week [*F*_(8, 179)_ = 11,208; *p* < 0.001]. *Post-hoc* tests revealed, as can be seen in Figure 5, that males showed a statistically significant preference for females in all weeks except week 1.

### Voxel intensity analysis

#### SIM

**Figure 6:**
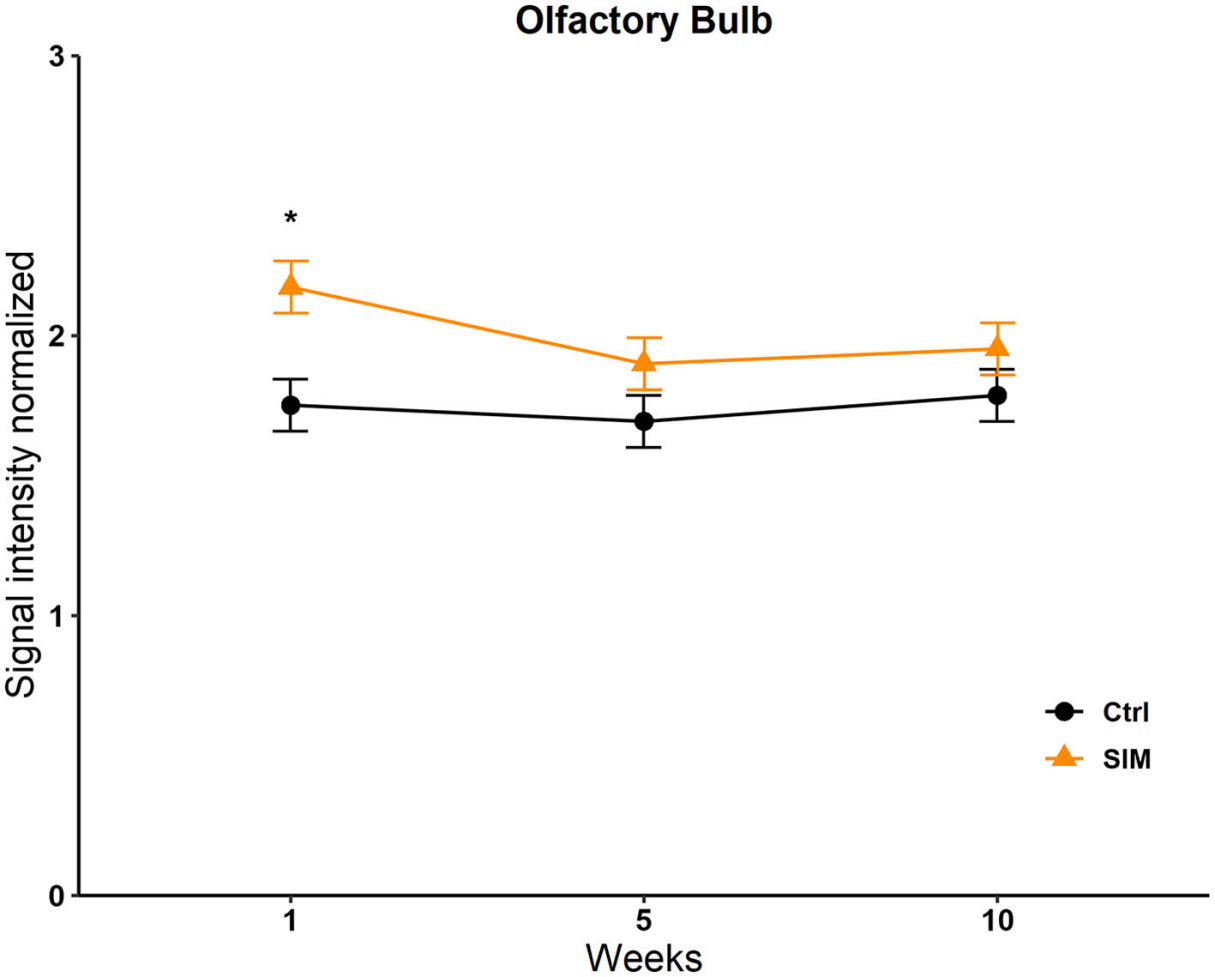
Signal intensity of the olfactory bulb (OB) in the SIM test. Data are expressed as mean ± SEM. *Different from the control group, *p* < 0.05.

Data from the statistics map of the SIM were analyzed by a two-way repeated-measures ANOVA and *post-hoc* Tukey's test. No differences were found in the BNST, the MPOA, the VMH, the NACC, the AMG, the STR, the VTA, and the Hipp; see Supplementary Table 5. We only found significant differences in the OB in the group factor [*F*_(9, 646)_ = 47; *p* < 0.017]. The *post-hoc* test revealed a significant increase in signal intensity in week 1 in the SIM group vs. the control group (*q* = 4,327, *p* = 0.006; Figure 6).

#### PP

**Figure 7:**
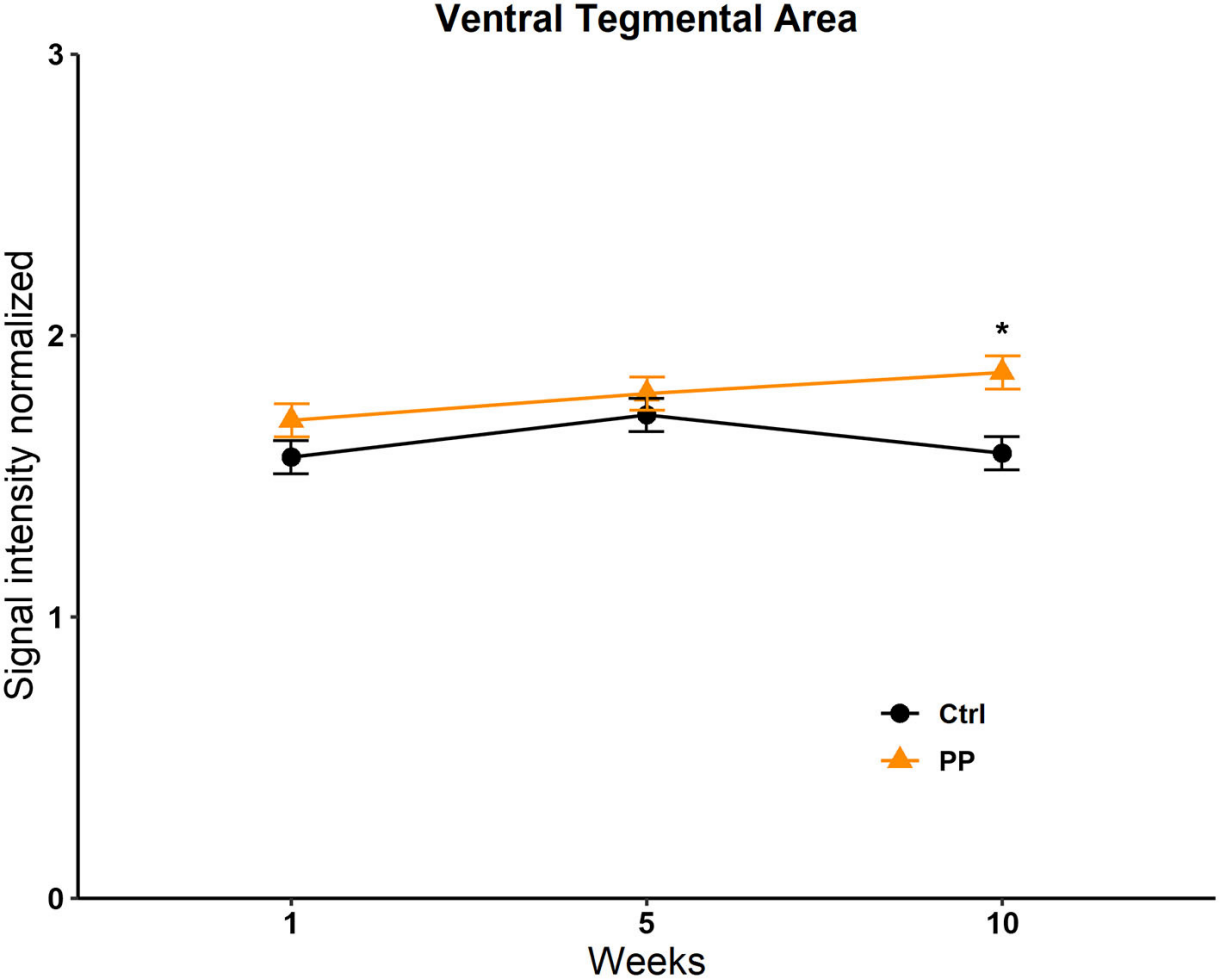
Signal intensity of the ventral tegmental area (VTA) in the PP test. Data are expressed as mean ± SEM. *Different from the control group, *p* < 0.05.

No differences were found in the OB, the BNST, the MPOA, the VMH, the NACC, the AMG, the STR, and the Hipp; see Supplementary Table 5. We only found a significant difference in the VTA in the group factor [*F*_(1, 13, 716)_ = 47; *p* = 0.008]. The *post-hoc* test revealed an increase in signal intensity in the experimental group vs. the control in week 10 (*q* = 5,681, *p* = <0.001; Figure 7).

### Circuits signal intensity

#### SIM group

We analyzed the signal intensity of the structures that form the socio-sexual behavior circuit (SBN) and the mesolimbic reward circuit (MRS). The SBN includes the OB, the BNST, the AMG, the MPOA, and the VMH. The MRS includes the NACC, the STR, the Hipp, and the VTA. We analyzed the changes along the weeks between groups with a two-way repeated-measures ANOVA and *post-hoc* Tukey's test. The factors were groups and weeks.

##### Socio-sexual behavior circuit

**Figure 8:**
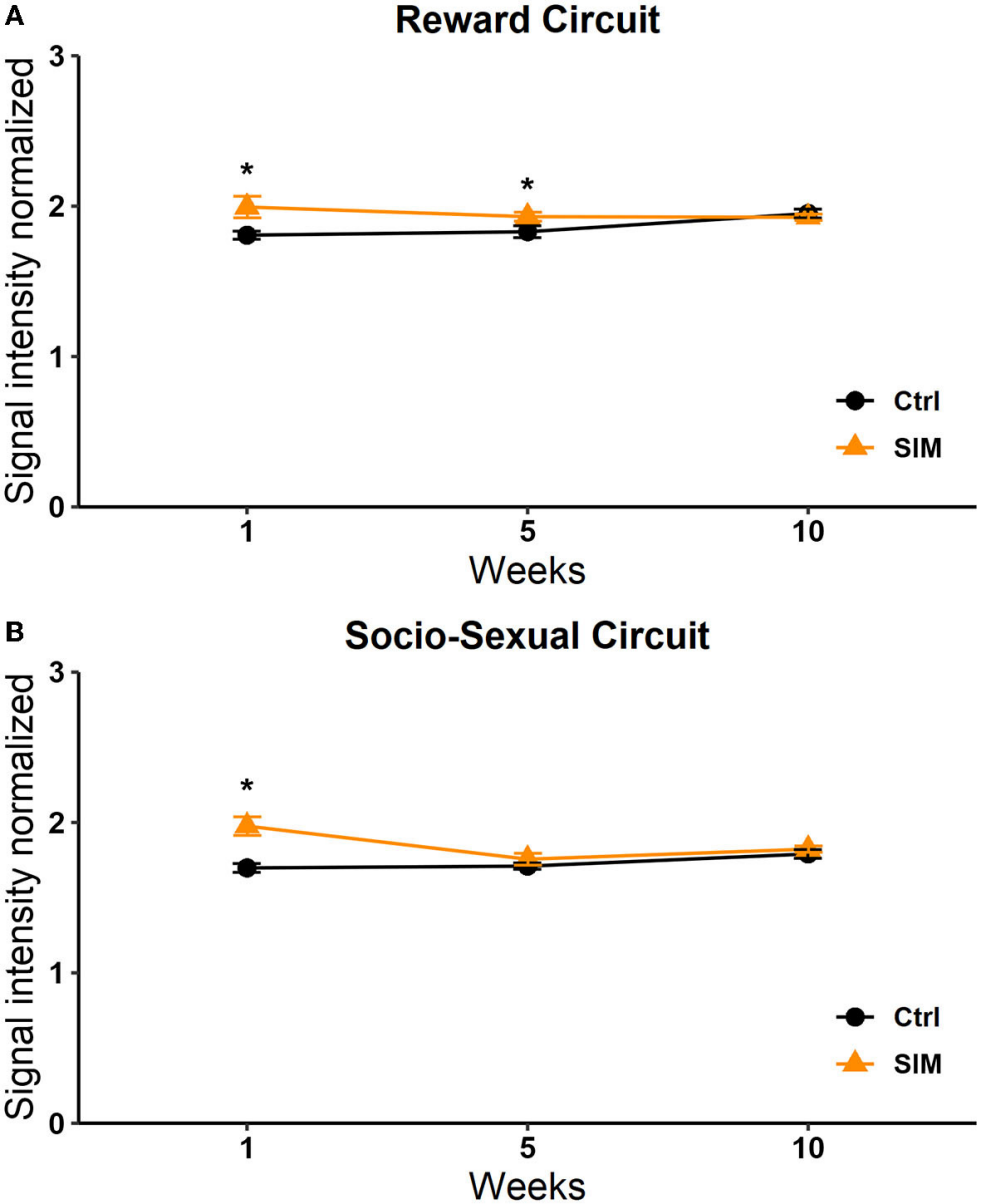
Signal intensity of the reward **(A)** and socio-sexual **(B)** circuit in the SIM test. Data are expressed as mean ± SEM. *Different from the control group, *p* < 0.05.

A significant effect was found in the group factor [*F*_(1, 264)_ = 11.468, *p* < 0.0008]. No effect was observed in the week factor [*F*_(2, 264)_ = 2.644, *p* = 0.073] or the interaction [*F*_(2, 264)_ = 2,642, *p* = 0.073]. The *post-hoc* Tukey's test revealed a significant increase in signal intensity in the SIM group vs. the control group in session 1 (*q* = 2,784, *p* < 0.05; Figure 8).

##### Reward circuit

A significant effect in the interaction was observed [*F*_(2, 210)_ = 4,115, *p* = 0.018]. No significant effects were observed in the week [*F*_(2, 210)_ = 1,258, *p* = 0.286] or group [*F*_(1, 210)_ = 2,590, *p* = 0.109] factors. The *post-hoc* Tukey's test revealed a significant increase in signal intensity in weeks 1 and 5 in the SIM group vs. the control group (*q* = 3.338, *p* = 0.005; Figure 8).

#### PP group

##### Socio-sexual behavior circuit

**Figure 9:**
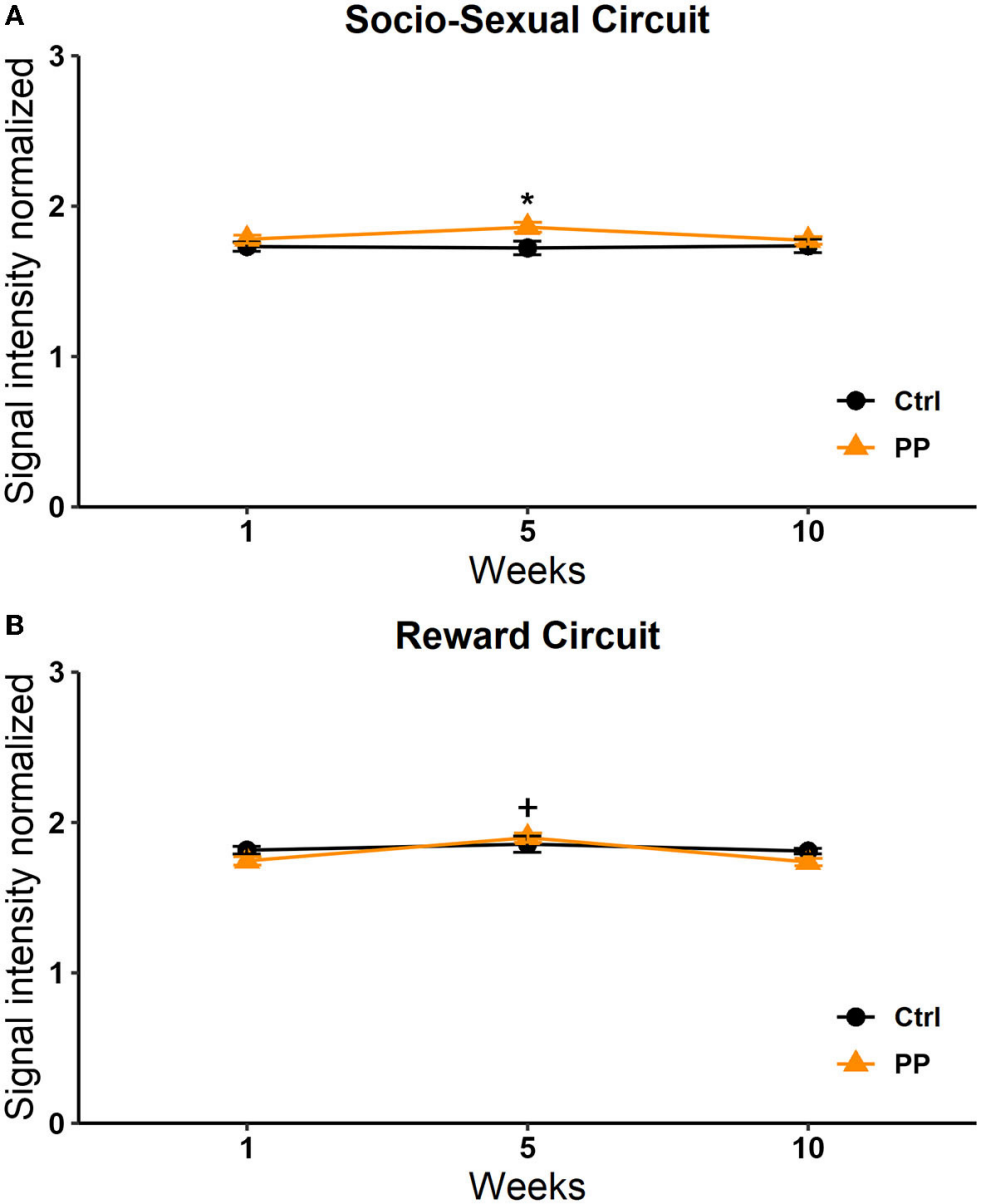
Signal intensity of the socio-sexual **(A)** and reward **(B)** circuit in the PP test. Data are expressed as mean ± SEM. *Different from the control group, *p* < 0.05.

A significant effect was found in the group factor [*F*_(1, 264)_ = 6,801, *p* = 0.010]. No significant effects were found in the week factor [*F*_(2, 264)_ = 0.956, *p* = 0.386] or the interaction [*F*_(2, 264)_ = 1.296, *p* = 0.275]. *Post-hoc* tests revealed a significant increase in signal intensity in week 5 in the PP group vs. the control group (*q* = 2.784, *p* < 0.05; Figure 9).

##### Reward circuit

With respect to the reward circuit, the two-way RM ANOVA analysis showed significant differences in the week factor [*F*_(2, 210)_ = 3.133 *p* = 0.046] and the interaction [*F*_(2, 210)_ = 3.495 *p* = 0.032]. No differences were observed in the group factor [*F*_(1, 210)_ =3.552 *p* = 0.061]. The *post-hoc* analysis revealed a significant increase in the PP group in week 5 vs. 1 (*q* = 2,787, *p* = 0.05) and week 5 vs. 10 (*q* = 3.338, *p* = 0.05; Figure 9).

### Cohen's *d* analysis statistics maps

**Figure 10:**
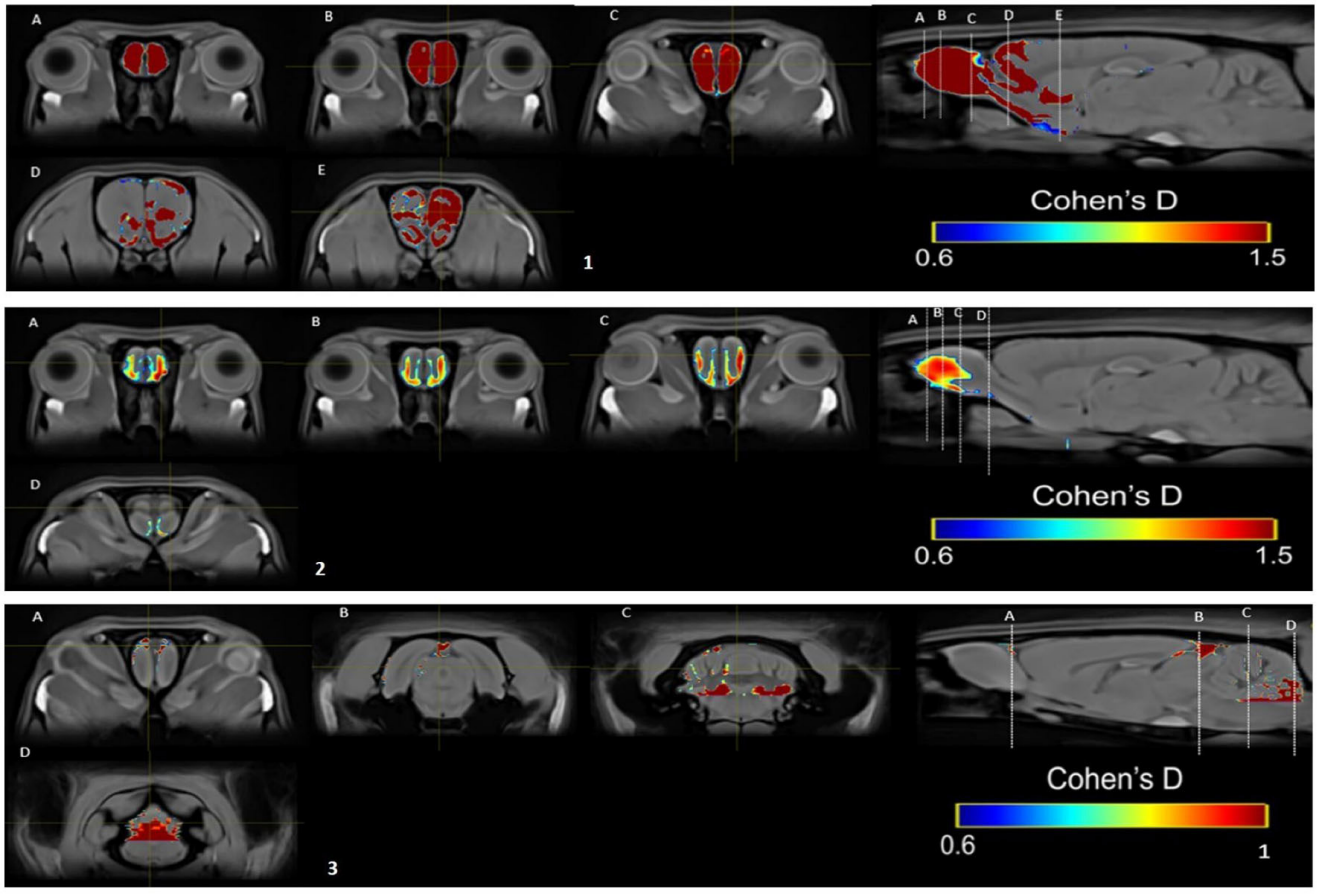
Results of the Cohen's *d* analysis comparing the SIM vs. control group in week 1 **(top panel)**, in week 5 **(middle panel)**, and in week 10 **(bottom panel)**.

Cohen's *d* analysis evaluates the effect size, comparing the differences between the two groups (Lakens, 2013). We compared the SIM vs. control group in weeks 1, 5, and 10 and the differences between weeks in the same group. Differences were found between groups in week 1 (Figure 10) and week 5 (Figure 10) large size changes (*d* = 0.6–1.5), and in week 10, medium-size effect (*d* = 0.6–1; Figure 10). These changes were observed in the OB in weeks 1 and 5. In week 10, we see the effect of size on OB and cerebellum. We did not find differences between weeks.

**Figure 11:**
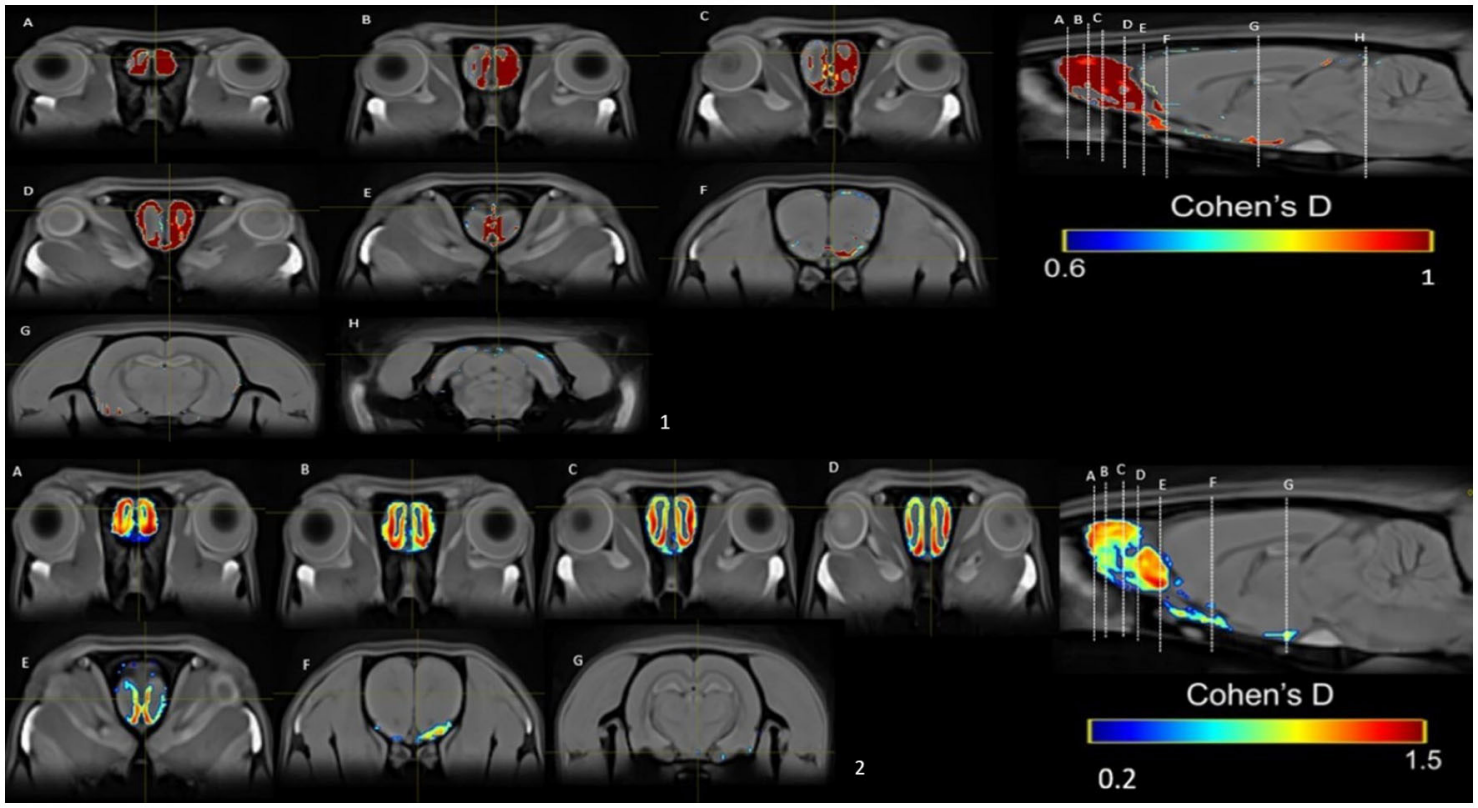
Results of the Cohen's *d* analysis comparing the PP vs. control group in week 1 **(top panel)** and in week 5 **(bottom panel)**.

**Figure 12:**
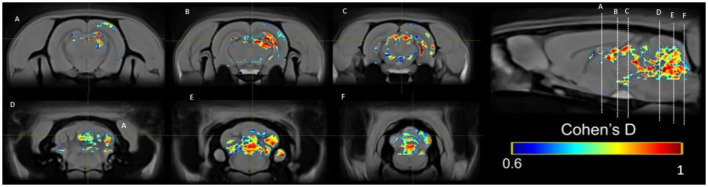
Results of the Cohen's *d* analysis comparing the PP group in week 5 against week 1.

**Figure 13:**
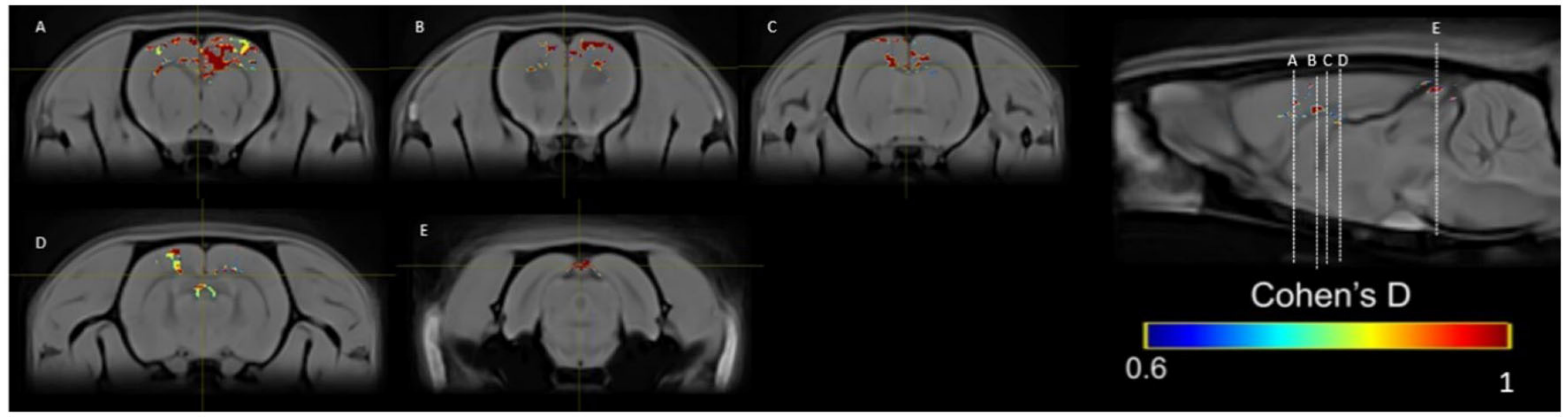
Results of the Cohen's *d* analysis comparing the PP group in week 5 against week 10.

In the PP group, we found a medium effect (*d* = 0.6–1) in the OB vs. the control group in week 1 (Figure 11). In week 5, we can see a large effect size (*d* = 0.2–1.5) in the BO and the MPOA (Figure 11). Between sessions, we observe a medium effect size in weeks 5 vs. 1 (Figure 12). Different brain areas were activated in anterior and middle brain structures. In weeks 5 vs. 10, we found a medium effect size on the cerebral cortex (Figure 13).

These results reflect a strengthening in the communication between structures that form the SBN and MRS circuits and other brain regions. As more sexual experience is acquired, a higher signal intensity is observed in the SIM group, indicating that when the subjects have more sexual experience, sexual motivation is a potent stimulus to induce higher activation in week 10. In the PP group, we found the highest activation in week 5, maybe because the execution of sexual behavior reached a threshold and no more activation could be observed in the latter weeks.

## Discussion

The interest in using MEMRI to study functional-specific biological processes is increasing. It has become clear that the rich biological characteristics of Mn^2+^ and its potent MRI relaxation properties are leading to exciting new opportunities to probe biological processes in animal models. In the present study, we demonstrated that 16 mg/kg is an excellent dose to obtain good contrast images without producing unspecific effects that could interfere with the expression of motivated behaviors. We observed a reduction in the distance traveled on the running wheel the day the subjects were injected with MnCl_2._ However, no significant effects were observed in sexual behavior or the rotarod test after MnCl_2_ administration. Previous studies have shown that the rotarod test is a susceptible test to identify motor alterations that induce unspecific effects, which could interfere with the display of sexual behavior; whenever motor execution is altered (evaluated by the rotarod), sexual behavior is also affected (Agmo et al., 1987). On the other hand, alterations in locomotor activity do not correlate with alterations in sexual behavior (Agmo et al., 1987). In the present experiment, MnCl_2_ administration did not affect the motivated behaviors tested. These results confirm our previous observations in female rats where the same dose of manganese did not affect running wheel, rotarod, or sexual behavior (Aguilar-Moreno et al., 2022), ratifying that the 16 mk/kg dose of manganese can be used to evaluate neuronal and circuit activation after a particular behavior and how this activity is modified as subjects acquire experience.

In the present study, we wanted to assess how the activation of brain regions of the SBN and the MRS circuits changes as subjects develop experience in the SIM and the PP tests. The male subjects preferred the female stimulus animals in both the SIM and PP tests, except for week 1, where no statistically significant differences were observed. After week 1 and as testing was extended, subjects increased their preference for the female in both tests, showing a systematic increase in the time spent with the female.

We found an increase in signal intensity in the OB in the first week of testing in the group of males tested in the SIM test compared to the control group. Cohen's analysis revealed that the effects were large. The OB processes volatile and non-volatile odors, sending the information to different regions of the SBN circuit. The OB is a laminar structure located anterior to the rodent forebrain and is the first relay station for olfactory information (Brunert and Rothermel, 2021). The accessory olfactory bulb (AOB) sends information mainly to the medial amygdala, which is highly involved in sexual behavior in male rodents. The AOB and the MeA also project to the MPOA (Hull and Rodríguez-Manzo, 2009a) and other regulatory nuclei of sexual behavior, such as the BNST and the VMH (Baum and Bakker, 2013). It is well-established that bilateral bulbectomy alters sexual behavior (Edwards et al., 1990). The OB is well-positioned for hormonal neuromodulation; some blood molecules can reach the OB more easily compared with other brain areas since the density of the blood capillary network, especially in the granular layer (GL), is very high (Lecoq et al., 2009). The OB is crucial in detecting sex steroid hormones (Baum and Bakker, 2013; Brunert and Rothermel, 2021). These hormones modulate odor production in males, being androgen-dependent. For example, sex differences in the chemical composition of rat urine have been attributed to the presence in males of preputial glands, which are organized during fetal life in response to the action of testosterone (Brouette-lahlou et al., 1991). Lactating mother rats spend more time displaying anogenital licking of male vs. female pups in response to pheromones from the preputial glands that incorporate into the male's urine (Moore and Samonte, 1986). These are examples of how the effects of sex hormones can induce a positive hedonic value, predicting the induction of a reward state, which leads to a higher activation of the OB.

We also found a significant increase in signal intensity in the socio-sexual circuit in the first week of testing in the SIM group compared to the control group. As described in the introduction, the SBN includes the OB, the AMG, the BNST, the MPOA, and the VMH. These brain regions play different roles in controlling male sexual behavior in rodents. For example, the MOB and the AOB send direct pheromonal information to the meAMY, and there is evidence suggesting that this brain region is involved in the reinforcing value of sexual pheromones (Lanuza et al., 2008). Inputs from the vomeronasal system are processed by the medial and cortical posteromedial amygdala via unidirectional projections (Scalia and Winans, 1975; Sah et al., 2003). The meAMY, in turn, projects mainly to the hypothalamus through the stria terminalis to modulate reproductive, aggressive, and parental behaviors (Canteras et al., 1994; Risold et al., 1997; Swanson and Petrovich, 1998). In addition to many other neurochemical markers, the meAMY is rich in steroid hormone receptors (Rasia-Filho et al., 2012), a defining characteristic of nodes in the social behavior network.

The role of the MPOA in the control of male copulatory behavior has been the subject of intense investigation. This brain region plays a crucial role in the control of male sexual behavior in every mammalian species studied to date. A substantial amount of data suggests that lesions of the MPOA/AH reduce sexual motivation (Paredes and Baum, 1997; Paredes, 2003). For example, male rats with bilateral destruction of the MPOA/AH show less preference for a receptive female than control males (Paredes et al., 1998). Moreover, the infusion of lidocaine inhibits male sexual behavior and reduces the time spent in the incentive zone of the stimulus female in a sexual incentive motivation test, a classic test of sexual motivation (Hurtazo et al., 2008). An early study demonstrated that male rats with lesions of the MPOA continued to lever press to obtain the presence of a receptive female, suggesting that the MPOA is involved in the consummatory aspects of male sexual behavior (Everitt and Stacey, 1987). As discussed, the study is difficult to interpret because bar-pressing was maintained by second-order reinforcement, making the results difficult to interpret. Previous reviews have detailed different evidence using a variety of methodologies demonstrating the involvement of the MPOA in the motivational aspect of sexual behavior (Paredes and Baum, 1997; Paredes, 2003).

The VMH is rich in sex steroid receptors (Holmes et al., 2008) and is highly interconnected with the amygdala and other regions of the hypothalamus. Similarly, by having a connection with the pituitary gland, it helps control endocrine hormonal secretion and responses to stress and reproduction (Xie and Dorsky, 2017). It participates in reproductive behavior and receives projections from nuclei related to the amygdala's olfactory system, such as the MeAMY (Sah et al., 2003). Recent electrophysiological recordings in freely interacting mice demonstrated that VMH neurons are activated by proximity to a pure olfactory cue, male urine (Falkner et al., 2016).

Our results demonstrate that the SIM test induced a higher signal intensity in the MRS in weeks 1 and 5 compared to the control group. The MRS includes the NACC, the STR, the HIP, and the VTA (O'Connell and Hofmann, 2011). Previous pharmacological studies reported that DA increased significantly in the NACC when the males were presented with a sexually receptive female behind a screen and increased further during copulation but not during locomotor activity (Damsma et al., 1992). The NACC facilitates copulatory behavior expression in sexually experienced male rats and is essential in appetitive behaviors for sexual reward (Everitt, 1990). It also participates in associative learning that links cues or contexts with rewards according to inputs from the amygdala or hippocampus (Cador et al., 1989; Everitt et al., 1991; Ito et al., 2008).

The ventral striatum (VS) is implicated in reward processing, participating in the computation of reward prediction errors (O'Doherty et al., 2003) and in the anticipation of reward delivery (Knutson et al., 2001). The VS has also been consistently implicated in the affective processing of rewarding stimuli (Cardinal et al., 2002; Delgado et al., 2004; Wang et al., 2016). The hippocampus (HIP) is essential for the memory representation of the environment or experiences (Bird and Burgess, 2008; Cascella and Al Khalili, 2023). As already described, in the case of sexual behavior, the approach behavior is intrinsically rewarding and is a measure of preference. If a stimulus is rewarding for the individual, they will remember the location of the rewarding stimulus, in this case, a potential sexual partner (Paredes and Ågmo, 2004). Another structure of the MRS is the VTA. The VTA is the primary source of the mesocorticolimbic dopaminergic tract and is considered a substrate of reward and locomotor behavior (O'Connell and Hofmann, 2011). Microinjection of dopaminergic agonists into the VTA of male rats delays the onset of copulation, presumably by stimulating autoreceptors on the cell bodies of the mesocorticolimbic dopamine tract. This stimulation reduces these neurons' firing rate, impairing locomotion and/or motivational processes (Hull et al., 1991).

Concerning the SIM group, our results suggest that sexual motivation is the strongest signal to activate both circuits in week 1 because sexual motivation without sexual experience activates the structures of the two circuits, which are complementary and integrate roles regulating vertebrate socio-sexual behaviors (O'Connell and Hofmann, 2011). Cohen's analysis revealed a large and medium effect size in the SIM group vs. the control group in the three sessions when we analyzed the complete brain activation. The OB seems essential during the acquisition of experience until week 5. However, in week 10, we observed a change in activation to caudal structures. The SBN and the RMS complement each other by regulating the evaluation of stimulus salience and behavioral output. During a sexual interaction, sexual motivation needs to induce an adequate level of sexual arousal to elicit penile erection and ejaculation. This anticipation of a possible sexual reward could explain the increase in the socio-sexual and reward circuits observed in sessions 1 and 5 of the SIM test.

In the PP test, the ROI analyses revealed a higher activation on week 10 in the VTA. The VTA is part of the reward circuit and one of the primary generators of DA in the brain. DA plays an important role in the regulation of male sexual behavior (Hull and Rodríguez-Manzo, 2009b) and it has been suggested that it improves sexual motivation and copulatory behavior (Beck et al., 2002) as well as genital reflexes (Hull et al., 2004). Another reason why we can see significant activation of the VTA in session 10 is because this structure is also related to movements during the execution of sexual behavior, that are displayed during the partner preference test.

We observed higher activation in week 5 in the PP test in both the SBN and MRS circuits. The structures of the SBN are reciprocally connected and contain receptors for sex steroid hormones, contributing to the display of sexual behavior (Hull et al., 1990; O'Connell and Hofmann, 2011). We also observed a higher activation in sessions 5 and 10 with respect to session 1 (when the subjects had no sexual experience) in the PP group. The PP group had a significant increase in signal intensity in weeks 1 and 5 compared to the control group. Cohen's analyses showed a significant increase and medium size effect in week 5 vs. weeks 1 and 10, in anterior and posterior structures, which perhaps reflects the threshold of experience, which does not change after 5 more weeks.

Sexual motivation during copulation guides behavior toward the satisfaction of sexual drive. Sexual arousal depends on the activation of brain networks, which control the behavioral and autonomic nervous system responses during sexual interaction and is mainly manifested by penile erection. In contrast, sexual motivation drives and maintains subsequent stages of sexual behavior (Agmo, 2011). The PP group showed on week 5 a significant increase in signal intensity, probably because they required sexual experience that they acquired in the first 5 weeks of testing, which was associated with an increase in the frequency of intromissions and ejaculations (Table 2).

**Table 2:** Sexual behavior parameters from subjects in the SIM group (*N* = 10) in weeks 2, 6, and 9 and the PP group (*N* = 10) in weeks 1, 5, and 10 (experiment 2).

**Groups**	**SIM (*****n*** = **10)**			**PP (*****n*** = **10)**		
**Sessions**	**2**	**6**	**9**	**1**	**5**	**10**
**Percentage**						
Mount	80	100	100	100	100	100
Intromission	80	90	100	60	90	100
Ejaculation	10	30	60	20	70	80^*^
**Number**						
Mounts	10.8 ± 3	24.1 ± 4.7^+^	23 ± 4.8^+^	0.75 ± 0.3	6.8 ± 1.3^*^	7.4 ± 1.8^*^
Intromissions	8.6 ± 3	20 ± 5.7	26.8 ± 5.7^+^	6.1 ± 1.4	16.1 ± 1.2^*^	15.5 ± 1.5^*^
Ejaculations	0.1 ± 0.1	0.3 ± 0.15	0.6 ± 0.16	1.5 ± 0.5	2.4 ± 0.4	2.9 ± 0.6^*^
**Latencies**						
Mounts	351 ± 114	97 ± 18.9^+^	125.6 ± 44.2	389.8 ± 85	97.1 ± 19.7^*^	79 ± 31.6^*^
Intromissions	622.8 ± 184	397.2 ± 133	295.2 ± 75.6	398.7 ± 82	129.7 ± 25^*^	126.1 ± 43.3^*^
Ejaculations	76.9 ± 76.9	262.4. ± 136	475.8 ± 155	351.5 ± 195	198.5 ± 43^*^	138.7 ± 84.4^*^
Post-ejaculatory interval	361.5 ± 16.4	341.3 ± 2.1	374 ± 22.8	324.8 ± 59.5	326.4 ± 34	321.3 ± 25.3

Data are expressed as mean ± SEM.

^*^Different from week 1 in the same group; p < 0.05.

^+^Different from week 2 in the same group; p < 0.05.

## Conclusion

The results of the present experiment further confirm that, under adequate doses, MEMRI can be used to evaluate brain regions and circuits activated by different motivated behaviors. SIM induces a higher signal intensity in the SBN and MRS in the first week compared to the PP group, which showed a significant increase until week 5. This activation is observed in the first week of testing. It is maintained until week 5, but only in the reward circuit, which indicates that sexual incentive motivation keeps this circuit active beyond the first contact with the female. The results of the whole brain analysis indicate that as the subjects acquire more sexual experience, different structures are activated.

## Data availability statement

The original contributions presented in the study are included in the article/Supplementary material, further inquiries can be directed to the corresponding author.

## Ethics statement

In accordance with the provisions of the Official Mexican Standard of SAGARPA, the Institute of Neurobiology has a Research Ethics Committee that is responsible for reviewing that studies and procedures involving human subjects, as well as the care and use of the Laboratory animals for research or teaching purposes are carried out within ethical and bioethical standards. NORMA OFICIAL MEXICANA (NOM-062-ZOO-1999). The study was conducted in accordance with the local legislation and institutional requirements.

## Author contributions

LG-T: Conceptualization, Investigation, Methodology, Project administration, Writing–original draft, Writing–review & editing. AA-M: Software, Methodology, Writing–review & editing. JO: Software, Writing–review & editing. SA: Methodology, Writing–review & editing. EA-C: Investigation, Writing–review & editing. RP: Conceptualization, Formal analysis, Funding acquisition, Supervision, Writing–original draft, Writing–review & editing.
