# Supplementary material for: Identification of neural circuits controlling male sexual behavior and sexual motivation by manganese-enhanced magnetic resonance imaging
Source: Front Behav Neurosci. 2023 Dec 21;17:1301406. doi: 10.3389/fnbeh.2023.1301406 (PMC10768062; doi:10.3389/fnbeh.2023.1301406)
Supplement: Supplementary file 1 [file Data_Sheet_1.pdf]

Sup. Table 1. Statistical results using the Friedman repeated measures ANOVA on sexual parameters comparing the different groups in experiment 1.

Groups	Control	MnCl2
Mounts		X^2 = 2,923 p=0.232
Intromissions	X^2 = 3,630 p=0.192	X^2 = 0,69 p=0.867
Ejaculation Latency	X^2 = 6,333 p=0.052	X^2 = 0,250 p=0.967
Postejaculatory Interval	X^2 = 17,198 p=0.055	X^2 = 4,750 p=0.120

Sup. Table 2. Statistical results using the Mann-Whitney Rank Sum Test on the sexual parameters in the two different groups in experiment 1.

**Weeks**	1	5	10
**Mounts**	MWU=27, T=82, p=0.086		MWU=47, T=117, p=0.615
**Intromissions**	MWU=20, T=72, p=0.361	MWU=17, T=80, p=0.096	MWU=43, T=112, p=0.620
Mounts Latency	MWU=28, T=88, p=0.307	MWU=34, T=121, p=0.211	MWU=33, T=88, p=0.212
Intromission Latency	MWU=22, T=69, p=0.341		MWU=48, T=103, p=0.940
Ejaculation Latency		MWU=50, T=105, p=0.90	MWU=43, T=88, p=0.903
Postejaculatory Interval	MWU=14, T=61, p=0.141	MWU=30, T=51, p=0.802	MWU=42, T=87, p=0.621

Sup. Table 3. Statistical results using the Friedman repeated measures ANOVA on the sexual parameters comparing the two groups in experiment 2.

**Groups**	SIM	PP
Ejaculations	X^2 = 4,0324 p=0.372	X^2 = 4,589 p=0.144
Ejaculation Latency	X^2 = 3,000 p=0.252	X^2 = 3,389 p=0.184
Postejaculatory Interval	X^2 = 4,667 p=0.194	X^2 = 35,678 p=0.301

Sup. Table 4. Statistical results using the Mann-Whitney Rank Sum Test on the sexual parameters in the groups from experiment 2.

Weeks	1	5	10
**Mounts**	MWU=50, T=105, p=0.90	MWU=43, T=88, p=0.903	MWU=42, T=87, p=0.621
**Intromissions**	MWU=35, T=81, p=0.688	MWU=27, T=72, p=0.164	MWU=29, T=126, p=0.120
**Ejaculations**	MWU=26, T=50, p=0.096	MWU=89, T=39, p=0.166	MWU=17, T=73, p=0.106
Mounts Latency	MWU=28, T=88, p=0.307	MWU=34, T=187, p=0.211	
Intromission Latency	MWU=29, T=71, p=0.798		MWU=26, T=81, p=0.076
Ejaculation Latency	MWU=14, T=61, p=0.141	MWU=30, T=51, p=0.802	MWU=43, T=88, p=0.903
Postejaculatory Interval	MWU=0,00, T=15, p=0.100	MWU=2, T=25, p=0.067	MWU=2, T=19, p=0.262

Sup. Table 5. Statistical results using the two way repeated measures ANOVA on analyzed ROIs in the two different groups in experiment 2.

Groups	SIM	PP
**OB**	S5 (q=2,218; p =0.149)<br>s10 (q=1,699; p=0.243)	[F(3,727) = 1,297; p = 0.05]
**NACC**	[F(0,197) = 3,9; p = 0.823]	[F(0,473)= 1,181; p = 0.683]
**MPOA**	[F(0,975) = 2,411; p = 0.401]	[F(1,534)= 1,101; p = 0.25]
**BNST**	[F(0,392)= 4,037; p = 0.683]	[F(0,0108)= 1,508; p = 0.989]
**VMH**	F(0,0443) = 4,079; p = 0.957]	[F(1,59) = 14,306; p = <0.078]
**HIPPOCAMPUS**	[F(1,0,408= 4,587; p = 0.673]	[F(,37) = 1,517; p = 0.698]
**AMG**	[F(0,982) = 6,995; p = 0.399]	[F(1,478) = 2,016; p = 0.262]
**VTA**	[F(0,385) = 3,939; p = 0.687]	S1 (q=1,988; p=0.175)<br>S5 (q=1,602; p =0.27)
**STRIATUM**	[F(0.691) = 3,3677; p = 0.517]	[F(0,945)= 1,103; p = 0.412]
